# Nucleic Acids in Human Glioma Treatment: Innovative Approaches and Recent Results

**DOI:** 10.1155/2012/735135

**Published:** 2012-05-21

**Authors:** S. Catuogno, C. L. Esposito, C. Quintavalle, G. Condorelli, V. de Franciscis, L. Cerchia

**Affiliations:** ^1^Istituto per l'Endocrinologia e l'Oncologia Sperimentale del CNR “G. Salvatore”, Via Pansini 5, 80131 Naples, Italy; ^2^Dipartimento di Biologia e Patologia Cellulare e Molecolare, University of Naples “Federico II”, Via Pansini 5, 80131 Naples, Italy

## Abstract

Gliomas are the most common primary central nervous system tumors with a dismal prognosis. Despite recent advances in surgery, radiotherapy, and chemotherapy, current treatment regimens have a modest survival benefit. A crucial challenge is to deliver drugs effectively to invasive glioma cells residing in a sanctuary within the central nervous system. New therapies are essential, and oligonucleotide-based approaches, including antisense, microRNAs, small interfering RNAs, and nucleic acid aptamers, may provide a viable strategy. Thanks to their unique characteristics (low size, good affinity for the target, no immunogenicity, chemical structures that can be easily modified to improve their *in vivo* applications), these molecules may represent a valid alternative to antibodies particularly to overcome challenges presented by the blood-brain barrier. Here we will discuss recent results on the use of oligonucleotides that will hopefully provide new effective treatment for gliomas.

## 1. Introduction

Glioma is the most common primary brain tumor, generally characterized by highly infiltrative nature, high malignancy, and poor clinical outcome. Despite great advances in surgical techniques, radiotherapy, and chemotherapy, the prognosis of this tumor remains poor [1, 2].

**Figure 1:**
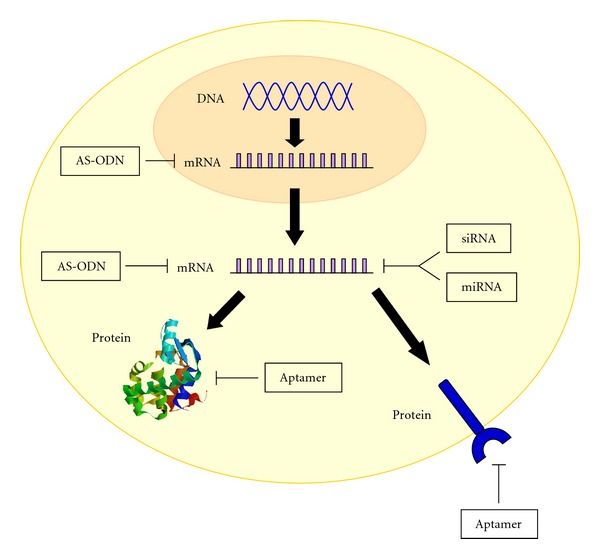
Mechanism of action of nucleic acids. AS-ODN, miRNAs, and siRNAs act on the mRNA preventing proteins translation. Nucleic acid aptamers fold into complex tridimensional shapes that allow direct binding to target protein.

Histologically gliomas are classified as astrocytomas, oligodendrogliomas, or ependymomas depending on cell morphology [3–7]. Genomic analysis of gliomas has revealed different subtypes that show distinct patterns of mutations, copy number alterations, and gene expression [8, 9]. On the basis of the grade of malignancy, as established by the World Health Organization [2], they can be further categorized as low grade (grade I and grade II) and high grade gliomas (grade III and grade IV). Grade I tumors are relatively benign and show the best prognosis. Grade II tumors contain some anaplastic cells and can progress to higher grade tumors. Grade III tumors show a high degree of anaplasia and mitotic activity and are often rapidly fatal. The most aggressive type of glioma is the grade IV astrocytoma or glioblastoma multiforme (GBM). This is a highly anaplastic and malignant tumor which is almost always fatal because of its resistance to radio—and chemotherapy. To date, antibody-based approaches have been developed for *in vivo *applications but, in most cases, adequate sensitivity has not yet been reached; they show toxicity *in vivo *and are not able to efficiently cross the blood-brain barrier (BBB). Promising alternative approach to antibodies is now represented by RNA and DNA oligonucleotides, including antisense (AS-ODN), microRNAs (miRNAs), small interfering RNAs (siRNAs), and nucleic acid aptamers (see Figure 1). Among them, only AS-ODNs are already in clinical development [10]. The most advanced is a phosphorothioate-modified AS-ODN (Trabedersen, AP 12009, Antisense pharma) directed against the transforming growth factor-*beta* 2 (TGF-*β*2), a protein that is massively produced by high-grade gliomas and promotes tumor cell proliferation, angiogenesis, invasion and metastasis. Thus, inhibiting TGF-*β*2 production, Trabedersen exerts multiple antitumor effects. To bypass the BBB, this AS-ODN has been administrated intratumorally using a single intratumoral catheter linked to a portable pump. On the basis of the positive results obtained in phase I and II clinical trials, in which Trabedersen resulted safe and well tolerated in patients with high grade gliomas, the oligonucleotide is currently in phase III [11].

Even if their clinical development is still being realized, miRNAs, siRNAs, and aptamers are emerging as innovative tools with an extraordinary potential for the treatment of different human diseases including glioma (see Table 1). In this paper we will focus on recent progresses and challenges of these three classes of oligonucleotides, with a major emphasis for those molecules that show great potential for clinical development.

## 2. Oligonucleotides versus Proteins

Oligonucleotides show many advantages over proteins as diagnostics and therapeutics. Indeed, they are entirely chemically synthesized, resulting in an easier production, in which complex manufacturing processes are avoided. Moreover, they can achieve high target selectivity, are sufficiently stable, and can be readily chemically modified to further enhance their stability, bioavailability, and pharmacokinetics. In this regard, the most effective modifications to reduce *in vivo* nuclease degradation are substitutions at the 2′-ribose of the pyrimidines with fluoro (2′-F-Py) or amino groups, but also introductions of 2′-O-Metyl purines [12–14], changes in the internucleotide linkages and in the nucleobases (such as the use of phosphorothioate) and capping at the oligonucleotide 3′-terminus have been successfully adopted [15]. Further, the use of locked nucleic acids, containing a methylene bridge to connect the 2′-O to the 4′-C, increases the stability of base pairing, stabilizing the duplex, and enhancing the resistance to nuclease [16–18]. The addition of polyethylene glycol (PEG) and other moieties can be used to increase oligonucleotides size enhancing their bioavailability and pharmacokinetic properties [19, 20].

One particularly attractive feature of oligonucleotides is that they are much less immunogenic than proteins. Indeed antibodies toward synthetic oligonucleotides are not generally produced and, the innate immune response by toll like receptors against nonself RNAs can be bypassed by the replacement of only uridines with their 2′-fluoro, 2′-deoxy, or 2′-*O*-methyl-modified counterparts [21–24].

## 3. Oligonucleotides Delivery to the Brain

A crucial challenge for human glioma treatment is to deliver drugs effectively to invasive glioma cells residing in a sanctuary within the central nervous system. Currently, it is not practical to administer drugs to humans by invasive procedures such as intracerebroventricular infusion or intracerebral injection, on the other hand, noninvasive intravenous administration of brain neurodiagnostic or neurotherapeutic agents remains a challenge because of the low permeability of the BBB. Indeed, the same mechanisms that protect the brain against intrusive chemicals can also frustrate therapeutic interventions. BBB is constituted by endothelial cells of brain capillaries which exhibit tight junctions that act as zippers and close interendothelial pores, thereby restricting the free movement of substances between the blood and the cerebral interstitial fluid [25]. More than 98% of small-sized drugs, including oligonucleotides, do not cross the BBB. However, different strategies in which oligonucleotides are conjugated to a transport vector that crosses the BBB, by means of receptor-mediated transcytosis, have been reported. Several recent papers describe the possibility to apply the brain drug-targeting technology for the diagnosis or therapy of many brain disorders [26–28]. Peptidomimetic monoclonal antibodies (MAbs) that bind endogenous transport system within the BBB, such as the insulin receptor, the transferrin receptor (TfR), or the leptin receptor, have been used for targeting neuropeptides, siRNAs, or antisense agents through the BBB *in vivo*. Further, immunoliposomes have been generated carrying small hairpin RNA expression plasmids for RNA interference (RNAi) effect (see “siRNAs and gliomas”) [29]. The immunoliposomes are then engineered with PEG, which stabilizes their structure in circulation. The tissue target specificity is given by conjugation of PEG residues to MAbs that bind the endogenous transport system within the BBB. This strategy presents a promising solution to the DNA/RNA delivery obstacle. In this context, it appears a challenging goal to reformulate the receptor-mediated approach, and to substitute antibodies with synthetic small aptamers, by designing new and original chimeric molecules. As discussed below (“nucleic acid aptamers and gliomas”), with ultimate objective of introducing therapeutic enzymes across the BBB, aptamers against the extracellular domain of the TfR have been selected [30].

Moreover, nanoparticles, which represent a very promising and innovative approach, have been also proposed to effectively and safely deliver drugs to the brain. Importantly, they can be structurally modified to deliver a wide range of therapeutics, improve delivery efficiency, and reduce side effects. Several types of nanoparticles such as linear polymers, hyperbranched polymers, dendrimers, liposomes, and micelles have been synthesized or engineered and successfully developed as carriers for brain-specific drug delivery, imaging, and diagnosis [31–35].

Once crossed the BBB, a safe and efficient therapeutic agent for glioma has to specifically target cancer cells in order to avoid unwanted side effects. In this regard, a promising approach is based on the use of chimeric molecules consisting of nucleic acid aptamers, directed against target proteins specifically expressed on the surface of cancer cells, fused to therapeutic siRNAs or miRNAs. The aptamers function as specific recognition ligands to target cell population, allowing the siRNA/miRNA therapeutic effect only on this subset of cells and thus substantially reducing unwanted side-effects such as death of normal cells [36–38].

## 4. MicroRNAs and Gliomas

**Figure 2:**
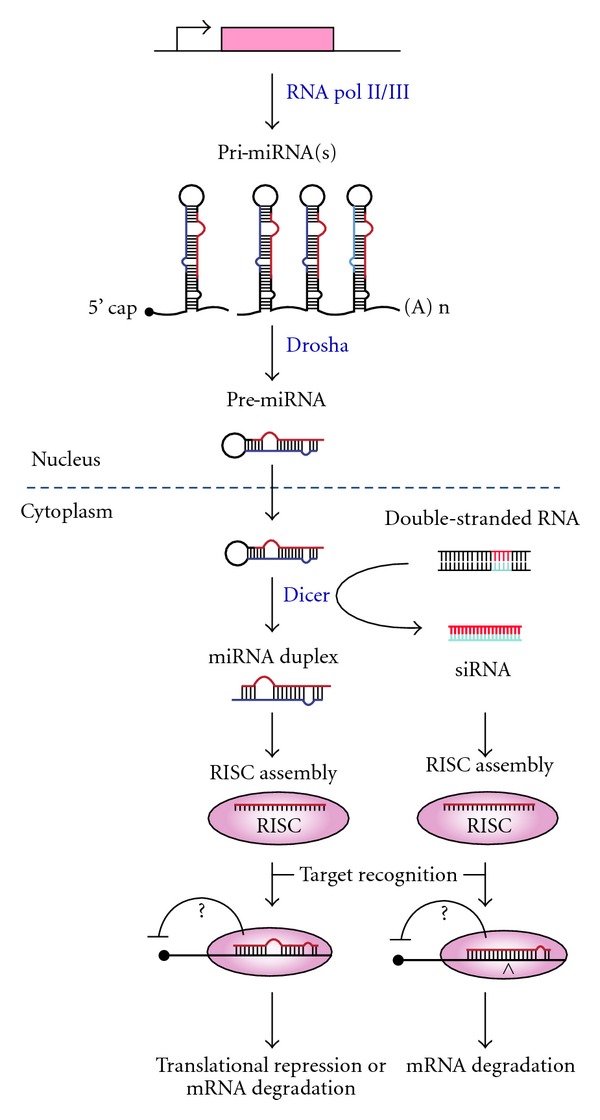
Scheme of mi/si RNA processing pathway. MiRNAs are first transcribed into long primary miRNAs (pri-miRNAs) by polymerase II or, in few rare cases, by polymerase III. Primary miRNAs are cleaved in the nucleus by an RNAse III enzyme, Drosha, inducing the conversion into precursor miRNAs (pre-miRNAs). Pre-miRNAs are transported into the cytoplasm by exportin-5 and subsequently processed by Dicer, a cytoplasmic endonuclease RNAse III enzyme, that generates a miRNA duplex. The functional strand of the mature miRNA is then incorporated into the RISC (RNA-induced silencing complex), that mediates the degradation or the translation inhibition of the target mRNA. For siRNA-mediated RNAi, double-stranded RNAs (dsRNAs) are chemically synthesized and then introduced into cell. dsRNAs are directly cleaved into small double-stranded siRNAs by Dicer and then incorporated into the RISC leading to target mRNA degradation.

MiRNAs are small noncoding RNA molecules (~20–25 nt), widely conserved through the evolution, that negatively regulate gene expression at posttranscriptional level in a sequence-specific manner. Following cellular processing (illustrated in Figure 2), they bind a specific site in the 3′UTR of a target mRNA allowing the degradation of the messenger if there is a perfect complementarity between the miRNA and the binding site in the 3′UTR, or translational repression in case of not perfect complementarity [39].

MiRNAs play an important role in cell cycle control, differentiation, proliferation, and apoptosis [40] and provide attractive prognostic biomarkers and therapeutic targets in different kind of cancer including glioma (see Table 2). Indeed, different miRNA expression levels have been observed in glioma tumors compared to surrounding normal brain [41, 42].

MiR-21 is overexpressed in different types of tumors, including glioma [43–45]. In glioblastoma it acts as an oncogene by suppressing apoptosis, thus suggesting that impaired apoptotic pathway associated with miR-21 overexpression could play an essential role in the pathogenesis of this kind of tumors. It has been reported that miR-21 is able to target different components of the p53, TGF-*β* and mitochondrial apoptotic tumor-suppressive pathways in glioblastoma. Indeed, proteins that stabilize p53 levels or that act as p53 transcriptional cofactors (such as p63, junction mediating and regulatory protein, topoisomerase I binding arginine/serine rich, tumor protein p53 binding protein 2, death-domain associated protein, heterogeneous nuclear ribonucleopreotein K), as well as TGF*β*R2/3 receptors, are directly targeted by miR-21, resulting in a failed activation of apoptosis and growth arrest [44]. In addition, miR-21 is able to regulate the invasive ability of glioma cells by targeting RECK (reversion inducing cysteine rich protein) and TIMP (tissue inhibitor of metalloproteinase-3), two well characterized inhibitors of matrix metalloproteinases proteins [46]. MiR-21 targets also programmed cell death 4, a tumor suppressor protein, involved in the inhibition of the translation and cell proliferation [47]. Remarkably, due to the high number of important proteins with tumor suppressive functions targeted by miR-21, it could be considered a major mediator in the pathogenesis of glioblastoma. Accordingly, its targeted downregulation in the more aggressive human glioblastoma, could represents a great therapeutic challenge.

Like miR-21, the cluster miR-221/222 was shown to be overexpressed in different cancers, including glioma [41, 45, 48]. The importance of the miR-221/222 cluster lies in the fact that it is able to impair cell cycle control leading to an increase of proliferation rate by targeting p27 [49]. Interestingly, it has been observed that CDK4 (cyclin-dependent kinase 4) is able to induce the expression of the miR-221/222, which in turn contributes to keep low p27 protein levels, thus stimulating continuous proliferation. Recently, it has been reported that miR-221 and miR-222 could contribute to glioma invasiveness targeting protein tyrosine phosphatase *μ*, a tyrosine phosphatase protein that suppresses cell migration and that is downregulated in glioblastoma [48].

Furthermore, miR-9 has been found highly expressed in cancer stem cells obtained from primary astrocytic glioblastomas [50] and in primary brain tumors [51]. This miRNA downregulates the RE1-silencing transcription factor (REST), promoting neural differentiation. REST, in turn, is able to repress miR-9, thus creating a negative feedback loop between a microRNA and a transcriptional regulator [52]. In addition miR-9 downregulates the tumor suppressor CAMTA1 (calmodulin binding transcription activator 1) which induces the expression of the antiproliferative Natriuretic Peptide type A [50].

On the other hand, several miRNAs have been reported to be underexpressed in glioblastoma samples compared to surrounding normal brain. MiR-124 and miR-137 represent two of the most significantly downregulated miRNAs in glioblastoma tissues [41]. Both of these two miRNAs directly target CDK6 that regulates cell cycle progression and differentiation [53]. MiR-7 is poorly expressed in human glioma tissues [54] and its overexpression in cultured glioma cell lines induces a reduction of viability and the ability to invade. This can be explained by the fact that the miR-7 is able to modulate the expression of epidermal growth factor receptor (EGFR) with the consequent suppression of Akt activity [55].

Another example is miR-128, that belongs to the class of brain-specific microRNAs [55]. As compared to normal tissue, the levels of this miRNA are extremely low in glioblastomas and, even at lesser degree, in low-grade gliomas [41, 56]. MiR-128 is able to decrease the expression of E2F transcription factor 3 (E2F3a) and of the BMI1 polycomb ring finger oncogene (Bmi-1) [56], and this may explain the ability of this miRNA to decrease cell proliferation both *in vitro* and *in vivo*.

Two studies have reported a downregulation of miR-181a and miR-181b in samples of human glioma cell lines [57]. The expression of these two miRNAs in glioma cells leads to the inhibition of growth and invasion, with induction of apoptosis [57]. However, molecular targets of these miRNAs remain still unknown.

Moreover, a recent report indicates that the expression profile of a set of seven miRNAs (miR-21, miR-128, miR-132, miR-134, miR-155, miR-210, and miR-409-5p) allows to discriminate between oligodendroglioma and glioblastoma [42]. Further, the alteration of miRNAs expression profile in gliomas has been found associated to their high resistance to the most commonly used antineoplastic agents [58–60], thus opening new prospective for glioma treatment.

## 5. siRNAs and Gliomas

siRNAs are synthetic double-stranded RNA molecules (~20–25 nt) that inhibit gene expression at posttranscriptional level in a sequence specific manner. As illustrated in Figure 2, the processing of siRNAs converges into the same molecular pathway of miRNAs mediating gene silencing [61].

SiRNAs have been successfully applied to the treatment of several human diseases [62] and in recent years this class of molecules has shown a great therapeutic potential for glioma [63].

To date, the most promising siRNA therapeutic application in glioma is represented by an interference RNA targeting tenascin-C (TN-C) [64, 65]. TN-C is an extracellular matrix protein that is overexpressed in different cellular processes including tumor growth, and its expression correlates with a higher tumor grade in human glioma [66].

 In a group of 46 patients affected by brain tumor [65], a dsRNA (ATN-RNA) complementary to the sequence of TN-C mRNA was administrated during surgery directly into neoplastic brain infiltration that could not be removed. Remarkably, the inhibition of TN-C synthesis induced growth tumor delay with improvements in survival and quality of life. Currently, this is the only example of clinical setting for siRNA-based therapies in glioma. However, a vast and increasing number of siRNAs targeting important molecular pathways, such as those involved in apoptosis, proliferation, cell invasion/migration, angiogenesis, and metabolism, has been studied and could represent good candidates for malignant glioma treatment (see [67] as review).

One particularly important drawback for the clinical development of siRNA therapeutics is the necessity of an appropriate and high efficiency *in vivo *delivery strategy to guarantee intracellular target accessibility and specificity of action. Different strategies for safe and efficacious siRNAs delivery to the brain have been proposed [68, 69] as summarized in Table 3. For example, a plasmid encoding a short hairpin RNA targeting the human EGFR was delivered to the brain through encapsulation in pegylated immunoliposomes (PILs), conjugated with two monoclonal antibodies allowing for the transport [29]. Indeed, the first antibody directed against the transferrin receptor enabled PILs to cross the BBB by transcytosis. Then, the antibody against the insulin receptor permits to specifically target PILs on glioma cells. Remarkably, weekly intravenous administration of PILs in mice bearing intracranial brain cancer caused an increase of survival. Furthermore, a directly coupling to TfR antibody via streptavidin-biotin linker was successfully applied for siRNA targeting to brain [70]. Other systems for siRNA delivery to brain include polyethyleneimine/siRNA complexes [71], coated nanoparticles resembling low-density lipoproteins [72, 73], and viral vector [74–78].

## 6. Nucleic Acid Aptamers and Gliomas

**Figure 3:**
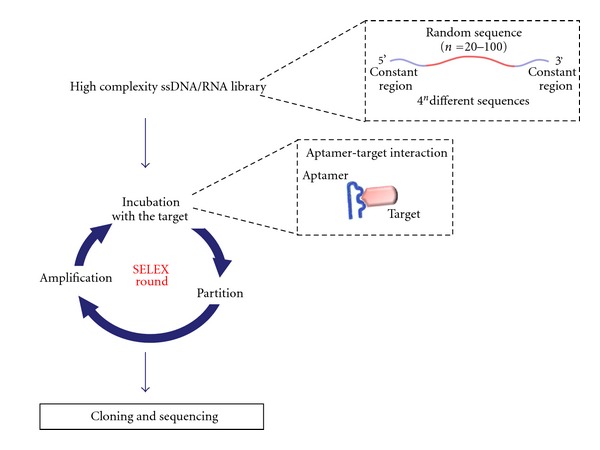
Scheme of SELEX technology. The starting point of the SELEX technology for aptamers production is the synthesis of a high complexity ssDNA/RNA library containing a variable region flanked by two constant regions. At each SELEX round the library is incubated with the target molecule and bound aptamers are recovered and amplified. At the end of the selection process, the PCR products are cloned and sequenced.

Aptamers are an emerging class of small (8–15 kDa) therapeutic nucleic acid molecules that unlike siRNAs/miRNAs, act by directly binding the target without interfering with its expression. They are generated by an *in vitro* procedure named SELEX that allows to isolate aptamers from combinatorial libraries through reiterated rounds of: (1) incubation of the library with the target molecule; (2) partition of unbound oligonucleotides from bound sequences; (3) dissociation of the aptamer-target complexes (4) amplification of the nucleic acids library enriched in sequences that bind to the target (see Figure 3). At the end of the selection process, the PCR products are cloned and sequenced and the best binding sequences are identified.

SELEX approach has been initially developed to generate aptamers against purified proteins and more recently the method has been extended to complex targets, including whole living cells, with the advantage of enabling the identification of aptamers that bind cell-surface specific antigens in their proper environments [36, 79]. To date, the list of aptamers generated against important diagnostic and therapeutic targets is growing rapidly and many of them are already in clinical trials for different human diseases [80–82].

As discussed above, TN-C is a very interesting target in glioma and aptamers directed against this protein have been generated by an approach based on a crossover SELEX experiment that involves crossing from cell-SELEX to protein- SELEX [83, 84]. Although, they have not been developed in therapy, they are very attractive tools for imaging. Indeed an RNA aptamer against TN-C, named TTA1, has been conjugated with ^99m^Tc and enables a clear imaging of TN-C-positive tumors in murine xenograft models of glioblastoma [85]. Another promising aptamer as diagnostic tool for glioma has been generated against transformed endothelial cells (EC) [86]. This aptamer was found to bind the rat homologue of mouse protein pigpen that regulates EC proliferation and is involved in angiogenesis. It has been shown that the anti-pigpen aptamer is able to selectively label microvessels of rat brain glioblastoma but not the vasculature of the normal rat brain and thus, it can be used to analyze pathological angiogenesis of glioblastoma.

Given the glioma complex cellular heterogeneity, the development of effective diagnostic and therapeutic approaches largely relies on the possibility to distinguish between even close tumor types with high accuracy. In this perspective, the development of the SELEX technology against living cells has a great potential. Indeed, this strategy allows to select aptamers able to specifically recognize diseased cells or discriminate different cell phenotypes. We have developed a differential cell-SELEX approach to isolate RNA aptamers against cell surface glioma-specific targets [87]. In such approach, that is a variant of cell-Selex that we used to generate aptamers against Ret [88], we performed iterative positive selection steps with human malignant U87MG cells, each preceded by counter-selection with non tumorigenic T98G cells. Seven different aptamers specifically recognize multiple targets on U87MG cells, which constitute a signature that might distinguish high-from low-or non-tumorigenic glioma cell lines and primary cultures. Furthermore, four out of the seven aptamers inhibit specific intracellular signaling pathways [87].

A key target for glioma treatment is the EGFR that is highly expressed in different types of cancer including glioma [89]. Recently, unmodified RNA-aptamers have been selected against the purified extracellular domain of EGFR. The aptamers have been conjugated to gold nanoparticles to specifically deliver these nanoparticles to EGFR-positive cancer cells by receptor-mediated endocytosis [90]. Further, glass surfaces functionalized with anti-EGFR aptamers have been used to capture with high sensitivity and specificity human and murine glioma cells expressing EGFR [91]. More recently, in order to improve *in vivo* aptamers stability, the same authors performed a new selection against a purified Fc-EGFR fusion protein using a 2′-F-Py RNA pool. They showed that one of the selected aptamers, E07, bound to the receptor as well as to A431 cells expressing EGFR, hampering EGF-dependent receptor autophosphorylation *in vitro* [92]. In addition, by cell-SELEX we generated a 2′-F Py-RNA aptamer that binds to EGFR with a binding constant of 10 nM. This aptamer inhibits EGFR-mediated signal pathways causing selective apoptotic cell death even in cells that are resistant to the most frequently used EGFR-inhibitors, as gefitinib and cetuximab. Remarkably, combined treatment with cetuximab and the aptamer shows clear synergy in inducing apoptosis and inhibiting tumor growth in a mouse xenograft model of human cancer, thus resulting in the first report of an anti-EGFR aptamer whose activity has been proved *in vivo *[93].

Activated deletion variant EGFRvIII is the most common EGFR mutation found in malignant gliomas. RNA aptamers have been recently generated against the histidine-tagged extracellular domain of EGFRvIII using an *Escherichia coli *expression system. These aptamers do not bind the protein expressed by eukaryotic cells likely because of glycosylation of the receptor [94]. Hence, EGFRvIII aptamers were transfected, where they act by binding the newly synthesized EGFRvIII and inhibit its glycosylation thus increasing the percentage of cells undergoing apoptosis.

Apart from the utility of aptamers as ligands of targets important for glioma, another promising application of aptamers against cell surface molecules is to employ their excellent targeting properties to specifically deliver therapeutic agents to the brain. Recently, with ultimate objective of introducing therapeutic enzymes across the BBB, aptamers against the extracellular domain of the TfR have been selected. These molecules were successfully used to deliver different reagents to mouse fibroblast cells [30] and can potentially be used, as alternative to antibodies, to design new and original chimeric molecules able to across the BBB through receptor-mediated transcytosis. In addition, aptamers showed an excellent potential as reagents for the delivery of miRNAs and siRNAs to a given target cell or tissue [36–38]. Even if to date there are no results for glioma, Giangrande's and Rossi's groups developed different innovative and effective chimeric molecules in which siRNAs were conjugated to aptamers targeting the prostate-specific membrane antigen or the HIV glycoprotein gp120, for specific aptamer-mediated siRNA delivery [95–99].

## 7. Conclusions

As discussed above, the interest for miRNAs, siRNAs and aptamers as innovative approaches for glioma treatment is rapidly increasing. Even if their clinical development is still being realized, oligonucleotides have several important advantages over traditional drugs and hold an extraordinary potential for glioma diagnosis and therapy. However, as with any novel therapeutic tool, further studies are required for their effective translation into clinic especially for what it concern glioma-specific tumor targeting. As discussed in this paper to address this issue, a highly intriguing approach could be the generation of chimeric molecules in which aptamers, directed against specific cell surface target proteins, are fused with therapeutic siRNAs or miRNAs.

Different approaches for aptamers-siRNA conjugation have been developed [36–38] and recently Archemix Corp., a leading biopharmaceutical company in the development of aptamers as therapeutics, started a collaboration with miRagen Therapeutics Inc., a biopharmaceutical company focused on developing innovative microRNA-based therapeutics for cardiovascular and muscle disease, for the development of conjugated aptamer-microRNA molecules capable of intracellular delivery and subsequent microRNA targeting.

##  Authors' Contributions

 C. Catuogno, C. L. Esposito and C. Quintavalle contributed equally to the work. 

## Figures and Tables

**Table 1:** Advantages and disadvantages of microRNAs, siRNAs, and aptamers.

	miRNAs	siRNAs	Aptamers
Advantages	(i) Chemically synthesized	(i) Chemically synthesized	(i) Chemically synthesized
	(ii) Small size	(ii) Small size	(ii) Small size
	(iii) Sufficiently stable	(iii) Sufficiently stable	(iii) High target selectivity
	(iv) Can be readily chemically modified	(iv) Can be readily chemically modified	(iv) High affinity
	(v) Less immunogenic than proteins	(v) Less immunogenic than proteins	(v) Sufficiently stable
		(vi) Already developed in clinical trials	(vi) Can be readily chemically modified
			(vii) Less immunogenic than proteins
			(viii) Already developed in clinic

Disadvantages	(i) Off-target effects (ii) Not yet developed in clinical trials	Off-target effects	Only a few aptamers as targeting agents for imaging

**Table 2:** MicroRNAs implicated in glioma.

miRNA		Function	Location	Targets	Cluster
Oncomir	miR-21	Inhibits apoptosis and promotes invasion	Chr 17 intergenic	TP53BP2 [44] DAXX [44] HNRNPK [44] RECK [46] TIMP3[46] PDC4 [47]	
	miR-221	Promotes proliferation and invasion	Chr X intergenic	PTP*μ* [48] P27 [49]	miR-221/222
	miR-222	Promotes proliferation and invasion	Chr X intergenic	PTP*μ* [48] P27 [49]	miR-221/222
	miR-9	Inhibits neural differentiation and induce proliferation	Chr 9 sense	CAMTA1 [50] REST [52]	

Tumor suppressor	miR-124	Induces G0/G1 cell cycle arrest; induce differentiation of adult mouse neural stem cells	Chr 8 sense	CDK6 [53]	
	miR-137	Induces G0/G1 cell cycle arrest; induces differentiation of adult mouse neural stem cells	Chr 1 sense	CDK6 [53]	miR-137/2682
	miR-7	Tumor suppressor; suppresses EGFR expression and independently inhibits Akt pathway	Chr 9 sense	EGFR [55]	
	miR-128	Inhibits cell proliferation by targeting Bmi-1 and E2F3a	Chr 2 sense	E2F3A BMI1 [56]	
	miR-181	Is downregulated and associated with poor prognosis	Chr 9 Sense antisense	unknown	miR-181a/b

**Table 3:** Strategies for siRNA delivery across the BBB.

Delivery system	siRNA target	Ref.
Pegylated immunoliposomes	EGFR	[29]
siRNA/TfR antibody complexes by biotin-streptavidin bridge	Luciferase	[65]
Polyethylenimine/siRNA complexes	Pleiotrophin	[66]
Nanoparticles/siRNA complexes	HIF-1*α*	[68]
Recombinant adeno-associated virus	Hec1	[69]
Herpes simplex virus 1	EGFR; Rad51	[70, 71]
Lentiviral vectors	shBcl-2 and S-TRAIL	[73]
